# Involvement of HLADQA1*05 in Patients with Inflammatory Bowel Disease Treated with Anti-TNF Drugs

**DOI:** 10.3390/medicina61010102

**Published:** 2025-01-13

**Authors:** Anna Pau, Ilaria Galliano, Elisa Barnini, Maddalena Dini, Antonio Pizzol, Alice Ponte, Stefano Gambarino, Pier Luigi Calvo, Massimiliano Bergallo

**Affiliations:** 1 Laboratory of Specialistic Pediatry, Department of Public Health and Pediatrics, School of Medicine, University of Turin, 10126 Turin, Italy; anna.pau@unito.it (A.P.); elisa.barnini@edu.unito.it (E.B.); maddalena.dini@unito.it (M.D.); massimiliano.bergallo@unito.it (M.B.); 2 BioMole srl. Via Quarello 15/A, 10100 Turin, Italy; stefano.gambarino@unito.it; 3 Unit of Pediatric Gastroenterology, Department of Pediatrics, Città della Salute e della Scienza di Torino, University of Turin, 10126 Turin, Italy; apizzol@cittadellasalute.to.it (A.P.); pontealice236@gmail.com (A.P.); pcalvo@cittadellasalute.to.it (P.L.C.)

**Keywords:** infliximab, adalimumab, IBD, HLA-DQA1

## Abstract

*Background*: Over the past decade, TNF inhibitors such as Infliximab and Adalimumab have become central to Inflammatory Bowel Diseases treatment, greatly enhancing patient outcomes. However, immunogenicity—where anti-drug antibodies diminish effectiveness—remains an issue, often requiring dose changes or combination therapies. Pharmacogenomics is increasingly applied in IBD to personalise treatment, especially since genetic factors like the HLA-DQA1*05 variant heighten the immunogenicity risk with IFX. This study aims to examine the relationship between the HLA-DQA1*05 variant and response loss or antibody development in patients regularly monitored on IFX or ADA. *Methods*: Sixty-five paediatric IBD patients were enrolled, with therapeutic drug monitoring (TDM) of IFX and ADA, conducted using immunoenzymatic assays. The presence of the HLA-DQA1*05 T>C allele variant was also tested using a Biomole HLA-DQA1 Real-time PCR kit. *Results*: The HLA-DQA1*05 rs2097432 T>C allele was present in 54% of patients on IFX and 69% of those on ADA. No statistically significant differences were found between HLA carriers and non-carriers across any of the three analysed groups: IFX, ADA and the overall anti-TNFα. *Conclusions*: Our study suggests that the HLA-DQA1*05 allele does not increase the risk of secondary loss of response to anti-TNF therapy, likely because most patients were on a combination of anti-TNF agents and immunomodulators, which can lower anti-drug antibody production. Testing for HLA-DQA105 can aid in personalising treatment and optimising therapy to minimise immunogenicity risks.

## 1. Introduction

Over the past decade, the treatment of inflammatory bowel diseases (IBD), which includes Crohn’s disease (CD) and ulcerative colitis (UC), has progressed significantly with the expansion of available drugs. Biologic agents, such as monoclonal antibodies targeting tumour necrosis factor (TNF), have become the gold standard in the treatment of inflammatory bowel disease (IBD). Infliximab (IFX), the first biologic therapy approved for IBD treatment, is a genetically engineered chimeric IgG1 antibody against human TNF-alpha. The later-approved Adalimumab (ADA) is a fully human monoclonal antibody that targets TNF-α. Both IFX and ADA bind to the transmembrane TNF-α, generating various intracellular signals that induce apoptosis, suppress cytokine production and arrest the cell cycle, leading to cell lysis via complement activation or via effector cells [1,2]. Approximately 30% of IBD patients do not initially respond to anti-TNF therapy (primary non-responders), and of those who do benefit, 40% to 50% experience a decline in efficacy within the first year, necessitating dose adjustments or a change in treatment, a phenomenon known as “secondary loss of response or immunogenicity” [1].

The aim of this study was to investigate whether the HLA-DQA1*05 variant rs2097432 is associated with loss of response and antibody development in IBD patients receiving IFX or ADA, with regular treatment monitoring. Molecular antibodies can drive immunogenicity by triggering the production of anti-drug antibodies, a process more common in patients treated with infliximab (IFX) than with adalimumab (ADA). This immunogenicity is a major contributor to reduced anti-TNF drug levels, infusion reactions, and the failure to achieve remission in Crohn’s disease.

The concomitant use of immunosuppressants (IMMs) such as thiopurines and methotrexate reduces the risk of immunogenicity, so TNF-α antagonists are often used in combination with IMMs. However, combination therapy is associated with an increased risk of long-term side effects, including lymphoma and serious infections [1,2,3,4]. Quantification of minimal drug levels after induction is associated with prolonged clinical response, as it identifies patients with inadequate IFX and ADA levels and optimises the individual dose [5,6]. Monitoring of the minimum concentration during maintenance therapy is useful to optimise the dosing regimen and improve therapeutic outcomes [7]. It has been shown that patients on maintenance therapy taking high doses of IFX are more likely to remain in remission than patients with undetectable minimal concentrations [8]. Low drug concentrations may be linked to the development of anti-drug antibodies due to the drug’s immunogenic properties [9].

Measuring these antibodies when drug levels are minimal or undetectable can assist in determining the appropriate therapeutic strategy. While TNF-alpha antagonists are highly effective in many cases, managing this loss of response remains a key challenge in long-term treatment [3,4,10,11]. Differences in patient response to treatment, which can be influenced by factors such as age, disease history and disease duration, account for only a small proportion of the overall variability observed between individuals. As a result, current research efforts are increasingly focused on investigating genetic variation to refine and improve personalised treatment strategies for IBD. This shift in focus has led to the search for new biomarkers that will allow more accurate patient stratification, taking into account the many factors that influence disease severity, drug response, adverse effects and overall prognosis [12,13].

Genetic testing is a key tool in identifying the most effective drug for each patient. “Personalised medicine” is now commonly equated with pharmacogenetics, which focuses on tailoring treatments based on an individual’s genetic profile. By analysing the connection between genetic variations, including polymorphisms, and differences in drug efficacy and toxicity, personalised medicine enhances treatment outcomes by ensuring that only those who are likely to respond receive the drug, while also reducing the risk of side effects in those more vulnerable. It is particularly useful in helping clinicians optimise therapeutic strategies for the treatment of IBD [13,14]. In 2019, Wilson et al. identified an association between the HLA-DQA1*05 variant, found in nearly 40% of Europeans, and an increased risk of antibody development against IFX, leading to a loss of treatment response and a higher rate of discontinuation. The mutation is carried by rs2097432 on chromosome 6 and the two most common HLA-DQA1*05 subtype alleles, HLA-DQA1*05:01 and HLA-DQA1*05:05, showed similar effects on the time to immunogenicity. Sazonovs et al. presented the PANTS (Personalised Anti-TNF Therapy in Crohn’s disease) study, a large, methodologically robust investigation. Their results showed that carrying this genetic variant was associated with a higher risk of immunogenicity and potential loss of response to anti-TNF treatment. Specifically, in the case of IFX monotherapy, 92% of patients with the variant developed antibodies after one year of treatment. However, this percentage was reduced when IFX was used in combination with immunomodulatory therapy (IMM). A similar pattern was observed with ADA, although the immunogenicity rates were lower with both monotherapy and combination therapy. In this study, HLA-DQA105 represents a group of allelic variants, each coding for a different protein: HLA-DQA105:01, HLA-DQA105:03 (A182S), and HLA-DQA1*05:05 (A11T, signal peptide). The HLA class II gene locus, which includes the HLA-DQA1 gene, is a complex region that encodes highly polymorphic, functional molecules responsible for presenting peptide antigens to T-helper cells. These cells stimulate B-cell maturation and antibody production. HLA class II molecules are heterodimers, consisting of an alpha chain (encoded by the A locus) and a beta chain (encoded by the B locus). The combination of a specific alpha and beta chain, as well as amino acid differences within and outside the peptide-binding groove, can influence protein trafficking and ligand binding [3,5,15]. Given the available evidence, we aimed to investigate whether the HLA variant is associated with a loss of response and antibody development in IBD patients receiving IFX or ADA with regular treatment monitoring.

## 2. Materials and Methods

### 2.1. Patients Population

#### 2.1.1. Cohort Characteristics

This study presents a monocentric, retrospective observational analysis of paediatric patients with IBD treated at the Pediatric Gastroenterology Unit of Regina Margherita Children’s Hospital in Turin, Italy. Patients included were under 18 years of age at diagnosis and received biologic therapy between February 2018 and June 2024. Demographic, clinical and biochemical information were obtained from the internal registry, anonymously. A total of 65 paediatric patients diagnosed with IBD were enrolled, of whom 49 received IFX, aged 4 to 18 years, while 16 were treated with ADA, aged 4 to 17 years, as detailed in Table 1.

#### 2.1.2. Samples Collection

For each patient treated with IFX and ADA, a blood sample in EDTA was collected, and from each, a tube containing 300 µL of blood and a tube containing 500 µL of plasma were prepared. The blood and serum tubes were stored in a freezer at −20 °C, until further analysis.

### 2.2. Serology Analysis

#### IFX, ADA and Antibodies Anti-Drug Quantification

Therapeutic drug monitoring (TDM) of IFX and ADA was performed using the immunoenzymatic assays RIDASCREEN IFX Monitoring and RIDASCREEN ADM Monitoring (R-Biopharm, Darmstadt, Germany), along with the detection of antibodies directed against the drug using the RIDASCREEN anti-IFX antibodies and RIDASCREEN anti-ADM antibodies kits (R-Biopharm, Darmstadt, Germany). Drug and antibodies concentrations were measured by spectrophotometric analysis using the 680XR microplate reader (Bio-Rad Laboratories (Milan, Italy). ELISA tests were performed on patient plasma according to the manufacturer’s instructions, both during the induction phase after the first administration and for monitoring subsequent administrations.

### 2.3. Genetic Analysis

#### 2.3.1. DNA Extraction

Genomic DNA was extracted from 300 μL of whole blood sample stocked at −20 °C using the Maxwell16 LEV Blood DNA kit (Promega, Milano, Italy) with the Maxwell16 System automated extractor (Promega, Milan, Italy) according to the manufacturer’s instructions. Automated DNA purification is based on sample lysis and DNA binding to paramagnetic particles as the primary separation principle, followed by multiple washes of the particle-bound molecules away from other cellular components [Maxwell 16 DNA purification kit technical manual TM284]. DNA concentration was measured with SimplyNano spectrophotometer (Biochrom, Holliston, MA, USA), diluted to a concentration of 10 ng/μL in elution buffer (Maxwell 16 lev Blood DNA kit, Promega, Milan, Italy) and stored at −20 °C until amplification.

#### 2.3.2. Dual Probes PCR Real-Time

The Biomole HLA-DQA1 Real-time PCR kit (BM-045) (Biomole, Turin, Italy) was used to test the presence or absence of the HLADQA1*05 T>C allele variant. The assay uses two probes, one specific for the native genotype and one for the variant, with two different fluorophores. This allows the simultaneous use of two probes in one reaction, enabling allelic discrimination analysis. Real-Time PCR was performed in a 96 well plate on CFX96 real time system in combination with the Bio-Rad CFX Maestro software ver. 1.0 (Bio-Rad Laboratories (Milan, Italy). Amplification reactions were performed in a volume of 20 μL containing 5 μL of DNA (50 ng in total) and 15 μL of amplification mix following the manufacturer’s instructions. The thermic profile amplification was as follows: 95 °C for 2 min allowing enzyme activation followed by 45 cycles of amplification at 95 °C for 15 s followed by 60 °C for 1 min.

#### 2.3.3. DNA Sample Sequencing

All the patient’s samples were sequenced to confirm the genotypes obtained with our PCR assay. PCR amplification was set up using 5 μL of DNA sample 10 ng/μL and 45 μL of amplification mix containing Mastermix GoTaq Hotstart Polymerse (Promega, Fitchburg, WI, USA), GoTaq Flexi Buffer 5× (Promega, Milan, Italy), MgCl 1.5 nM, dNTPs 5 mM, primer forward 5′-TGTGCACCTACCCTCACTTT-3′ and reverse 5′-CCCTTCAACCACAACCCTAT-3′ 1000 nM. Run was performed on Thermocycler at 95 °C for 2 min, 35 cycles at 95 °C for 15 s, 60 °C for 30 s and 72 °C for 30 s. PCR products were purified using Illustra ExoProStar (Sigma Aldrich, Darmstadt, Germany) adding 1 μL of Alkaline Phosphatase and 1 μL of Exonuclease I to 5 μL of amplification product and incubated at 37 °C for 15 min and 80 °C for 15 min. Sequencing reaction was set using 1 μL BigDye Terminator (Thermofisher Scientific, Waltham, MA, USA), 2 μL sequencing Buffer, 0.5 μL of Purificated DNA, Primer 2 μM forward 5′-TGTGCACCTACCCTCACTTT-3′ and reverse 5′- CCCTTCAACCACAACCCTAT-3′ and run in the thermocycler at 96 °C for 1 min and 28 cycles at 96 °C for 10 s, 50 °C for 5 s and 60 °C for 4 min. Sequences obtained from amplification were purified using magnetic beads and 4 μL of eluted amplicons were used for sequencing in the Applied Biosystem 3500 genetic analyser (Thermofisher Scientific, Waltham, MA, USA).

### 2.4. Statistical Analysis

Chi-Square Test or Fisher’s Exact Test were used to evaluate the association between antibody formation and patient genotype. Patients with one or two variant alleles were grouped together and compared to those with two normal alleles. For data collection and fold change calculation, Excel software was used. Statistical analyses were carried out using the Prism software (GraphPad Software, version 7, La Jolla, CA, USA). In all analyses, *p* < 0.05 was taken to be statistically significant.

## 3. Results

### 3.1. Patients

Demographical and clinical characteristics of enrolled patients are detailed in Table 1.

The conditions affecting patients treated with IFX included ulcerative colitis (UC) in 23 patients, Crohn’s disease (CD) in 23 patients, and very early-onset inflammatory bowel disease (VEOIBD) in 3 patients. Drug monitoring (measurement of circulating drug levels at regular intervals) was performed in all patients as proactive or reactive, with reactive measurement of levels only in case of loss of response. The median follow up period was 4.2 yrs (1.9–5.8), 2.8 yrs (1.5–5.2) and 5.7 yrs (2.8–10.4) for CD, UC and VEOIBD, respectively. The analysis of patient responses to IFX therapy revealed that 11 patients discontinued treatment, 1 showed no primary response, 6 lost responses to the drug, 2 experienced infusion reactions, 1 achieved remission, 35 required dose escalation, and 10 had to switch to an alternative medication. In the group of patients treated with ADA, six were diagnosed with ulcerative colitis, nine with Crohn’s disease, and one had very early onset IBD. Following treatment initiation, five patients discontinued treatment, none failed to respond, four lost responses to the drug, none had an infusion reaction, none are in remission, four required dose escalation, and five patients had to switch to another drug.

### 3.2. IFX and Antibodies Anti-Drug Quantification

Serum IFX levels and anti-drug antibodies levels were quantified, during the induction phase, after the first administration, and for monitoring subsequent administrations.

Serum levels of IFX were as follows (medians, IQR 25–75%): 7.54, 2.40–12.00 μg/mL. Anti-drug antibodies were revealed in three patients (6.5%) and the serum levels were as follows (medians, IQR 25–75%): 460.85, 295.90–690.55 ng/mL.

### 3.3. ADA and Antibodies Anti-Drug Quantification

Serum ADA levels and anti-drug antibodies levels were quantified, during the induction phase, after the first administration, and for monitoring subsequent administrations.

Serum levels of ADA were as follows (medians, IQR 25–75%): 9.75, 6.50–12.00 μg/mL. Anti-drug antibodies were revealed in one patient (6.25%) and the serum level was 109.7 ng/mL.

### 3.4. Genotyping of HLADQA1*05

Dual-probe real-time PCR was employed to define the HLA-DQA1*05 genotype in 49 patients treated with infliximab (IFX) and 16 patients treated with adalimumab (ADA), studying the presence of the T>C variant (rs2097432). In the cohort of total patients undergoing anti-TNF therapy, 28% exhibited both normal alleles (genotype TT), 43% carried both variant alleles (genotype CC), and 29% possessed one variant allele (genotype TC) (Table 2). The genotypic frequencies were TT 0.28, CC 0.43 and TC 0.29. The results of the genotypic test were used to calculate allelic frequency.

In patients treated with IFX, the HLADQA1*05 variant C allele was present in 54% of patients, while in those treated with ADA, the frequency was 69%. Considering the total of patients undergoing anti-TNF treatment, the variant allele was found with a frequency of 58% (Table 2). HLADQA1 rs2097432T>C was in Hardy–Weinberg equilibrium (*p* = 0.2176).

### 3.5. Association Between HLA-DQA1*05 Variant and Immunogenicity

The association between the development of anti-drug antibodies in patients receiving infliximab (IFX) and adalimumab (ADA) therapy and the presence of the HLA-DQA1*05 genetic variant was investigated. Analyses were assessed both with patients’ genotype (Table 3), and with HLA-DQA1 alleles (Table 3).

None of the IFX patients with the TT genotype had anti-drug antibodies, while one with the TC genotype and two with the CC genotype had anti-drug antibodies. In the cohort of patients treated with ADA only one patient with the CC genotype exhibited antibody formation (Table 4).

**Figure 1:**
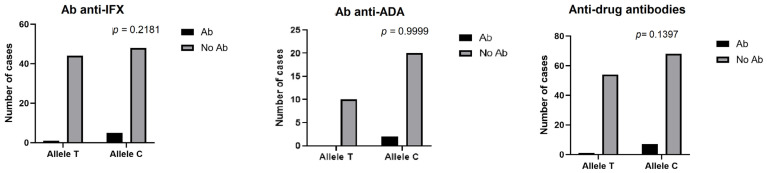
Association between allelic variant of HLA-DQA1*05 and development of anti-drug antibodies: anti-Infliximab (anti-IFX), anti-Adalimumab (anti-ADA) and overall anti-TNFα. Note: Ab = patients who developed antibodies; No Ab = patients who did not develop antibodies; anti-drug antibodies = Ab anti-IFX + Ab anti-ADA.

Our data showed no statistically significant associations between the normal or variant HLADQA1*05 allele and the immunogenicity against the therapy in the three groups: patients treated with Infliximab (*p* = 0.5499), patients treated with Adalimumab (*p* > 0.9999) and the overall group treated with anti-TNFα (*p* = 0.5685) (Figure 1).

**Figure 2:**
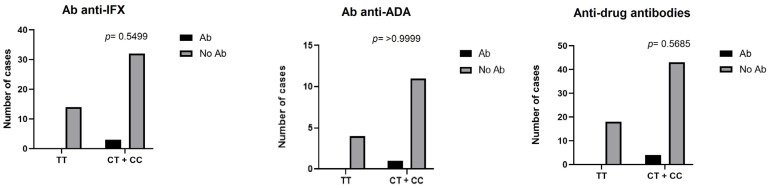
Association between genotypic variant of HLA-DQA1*05 and development of anti-drug antibodies. Note: Ab = patients who developed antibodies; No Ab = patients who did not develop antibodies; anti drug antibodies = Ab anti IFX + Ab anti ADA.

Furthermore, the association between the formation of antibodies and genotype was analysed. The results showed no statistically significant associations across any of the three groups: Ab anti-IFX (*p* = 0.5499), Ab anti-ADA (*p* > 0.9999) and Ab anti-TNFα (*p* = 0.5685) (Figure 2).

## 4. Discussion

Data from the literature show that around 30% of IBD patients initially do not respond to anti-TNF therapy, categorising them as primary non-responders. Among those who show an initial benefit, 40% to 50% experience a drop in treatment effectiveness within the first year, necessitating dose adjustments or a switch in therapy. This issue, known as “secondary loss of response” or immunogenicity, is more common with IFX than ADA. Immunogenicity plays a major role in reducing anti-TNF drug levels, triggering infusion reactions, and hindering remission, particularly in Crohn’s disease, making it a critical factor in the diminished response to biologic treatments [3,5,6]. Pharmacogenomic strategies are increasingly being applied in IBD to enhance drug safety and tolerability. A prime example is the use of TPMT genotyping to identify IBD patients at high risk for azathioprine-related myelotoxicity. Additionally, several studies have begun to emerge, indicating a link between genomic variations in the HLA gene region and drug-related adverse events [1,16]. In 2019, Wilson et al. found that the HLA-DQA1*05 variant rs2097432, present in nearly 40% of Europeans, is linked to a higher risk of developing antibodies against IFX. This response increases the likelihood of treatment failure and a higher discontinuation rate [1]. Sazonovs et al. conducted the PANTS (Personalised Anti-TNF Therapy in Crohn’s Disease) study, a large and methodologically sound investigation. Their findings indicated that possessing the specific genetic variant HLADQA1*05 increased the risk of immunogenicity and potential loss of response to anti-TNF therapy. Notably, among patients on IFX monotherapy, 92% developed antibodies after one year of treatment [5]. Based on this context, we examined the presence of the HLA-DQA1*05 T>C allele variant in 49 patients receiving IFX and 16 patients receiving ADA. This included drug monitoring and an analysis of the possible association between the variant and the development of anti-drug antibodies. In contrast to previous studies, the results of our study did not identify a statistically significant correlation between the presence of the HLA-DQA1*05 T>C allele variant and the formation of anti-drug antibodies in the three tested groups: IFX, ADA, and in the overall group of TNF antagonists. The negative outcomes of our study provide additional support for alternative explanations for the loss of response to anti-TNF therapy, as suggested by other research, particularly emphasizing the importance of serum drug levels during the induction phase. These factors may overshadow the impact of HLA on immunogenicity. The PANTS post hoc analysis conducted by Spencer et al., for instance, suggests that HLA carriers do not face an increased risk of developing antidrug antibodies, nor does HLA status affect drug level sustainability. Instead, response failure appears to be more closely related to serum drug concentrations. Thus, ensuring optimised dosing from the induction phase to achieve adequate drug levels may help lower the risk of immunogenicity [17,18]. Furthermore, another possible explanation for our findings is that almost all of our patients were treated with a combination of anti-TNF therapy and immunomodulators such as azathioprine, regardless of their genetic variant carrier status. Indeed, studies have shown that the addition of immunomodulators, including azathioprine, 6-mercaptopurine, or methotrexate, to anti-TNF therapy can reduce anti-drug antibodies production rates and enhance patient response to anti-TNF agents [19]. In support of this, a retrospective single-centre cohort study by Pascual-Oliver et al. involving IBD patients who received anti-TNF therapy as their first biologic treatment documented HLA-DQA1*05 status and found that carrying the HLA-DQA1*05 variant did not predict either primary or secondary response failure to anti-TNF treatment. In this study, as in ours, the majority of the analysed patients were treated with combo therapy with anti TNF and immunomodulators before their HLADQA1 status was determined [20]. However, the use of immunomodulators has been linked to a higher risk of infections in ulcerative colitis and malignancy in Crohn’s disease. Additionally, combination therapy further elevates the risk of serious and opportunistic infections, as well as an increased likelihood of lymphomas and nonmelanoma skin cancer [21,22].

The limitations of the present study, beyond the combination therapy employed, pertain to the modest patient cohort that was evaluated for the variant. Further studies, encompassing a more substantial number of patients treated exclusively with monoclonal antibodies, could prove instrumental in delineating the immunogenic risk associated with the variant in HLA-DQA1*05. Furthermore, proactive drug monitoring was utilised to adjust the administered dosage to the patient according to the measured concentration in the blood, which may have influenced the outcomes.

We still believe that, even though it does not align with our results, genotyping for rs2097432 may be instrumental in tailoring treatment decisions, aiding in the selection between anti-TNF monotherapy, combination therapy with an immunomodulator, or even alternative therapies with lower immunogenicity risks. Patients without the risk allele, especially those with previous adverse reactions to thiopurines or methotrexate, or those at high risk for opportunistic infections, may avoid the added risks of combination therapy and be well-managed with ADA or IFX monotherapy. For patients without identified genetic variants, the high negative predictive value suggests a low likelihood of developing immunogenicity on TNF-α antagonist monotherapy. Conversely, those who test positive for the HLA-DQA1*05 variant may benefit from combination therapy, or alternatively, proactive therapeutic drug monitoring (TDM) could help reduce the risk of immunogenicity.

Testing for HLA-DQA105 is also valuable when considering de-escalation from combination therapy and discontinuing immunomodulators. Patients without the HLA-DQA105 variant may proceed with de-escalation with greater confidence, while those with the variant may face a higher immunogenicity risk on TNF-α antagonist monotherapy. In such cases, continuing combination therapy is often preferable, but if de-escalation is pursued, proactive TDM may provide helpful ongoing support [2,5,6].

## 5. Conclusions

Our study indicates that carrying the HLA-DQA1*05 allele may not increase the risk of secondary loss of response to anti-TNF therapy; however, further studies on patients treated with ADA or IFX exclusively are needed.

A limit of this study is that almost all of our patients underwent combination therapy with anti-TNF and an immunomodulator, which helps to reduce the presence of anti-drug antibodies. Moreover, the cohort of patients was too small to draw definitive conclusions. Further studies are needed to explore the correlation between the HLA-DQA1 variant and the formation of anti-drug antibodies, enabling the use of this test as a useful biomarker for assessing immunogenicity. The integration of genetic testing for the HLA-DQA1*05 variant into the clinical approach to IBD management represents a significant advancement in the field. This approach combines the pharmacogenetic association with immunogenicity and the pharmacokinetic monitoring of anti-TNF-alpha agents, thereby enhancing the personalisation of therapy, and improving patients outcomes.

This development signifies a substantial advancement towards the personalisation of therapeutic interventions, integrating diverse methodologies and evaluations to optimise patient benefits. It facilitates the early identification of patients with an elevated risk of treatment failure and discontinuation rates.

## Figures and Tables

**Table 1:** Patients’ characteristics and treatment outcomes.

			IFX	ADA
Age	Median (IQR)		12.7 (11–15.25)	2.11 (10–15)
Total			49	16
Pathology	Ulcerative colitis	n. (%)	23 (47)	6 (38)
	Crohn’s disease	n. (%)	23 (47)	9 (56)
	Very early onset inflammatory bowel disease	n. (%)	3 (6)	1 (6)
Drug monitoring	Drug suspension	n. (%)	11 (22)	5 (31)
	No response	n. (%)	1 (2)	0
	Loss of response	n. (%)	6 (12)	4 (25)
	Infusion reaction	n. (%)	2 (4)	0
	Remission	n. (%)	1 (2)	0
	Escalation	n. (%)	35 (71)	4 (25)
	Drug’s change	n. (%)	10 (20)	5 (31)

Notes: IFX = infliximab, ADA = adalimumab, IQR = interquartile range (25–75%), n. = numerous, % = percentage.

**Table 2:** Genotyping and allelic distribution of HLADQA1 (rs2097432).

HLADQA1 (rs2097432)	IFX N. (%)	ADA N. (%)	ANTI-TNF N. (%)	ANTI-TNF FREQUENCY
Genotype TT	14 (29)	4 (25)	18 (28)	0.179
Genotype CT	17 (35)	2 (12.5)	19 (29)	0.488
Genotype CC	18 (37)	10 (62.5)	28 (43)	0.333
Allele T	45 (46)	10 (31)	55 (42)	0.423
Allele C	53 (54)	22 (69)	75 (58)	0.577

Notes: TNF = tumour necrosis factor, n. = numerous, % = percentage, Anti-TNF = IFX + ADA.

**Table 3:** Contingency table: association between genotypes of patients treated with IFX and ADA and the presence of anti-drug antibodies to anti-TNFα.

HLADQA1 (rs2097432)	IFX			ADA			ANTI-TNF		
	Ab	No Ab	TOT	Ab	No Ab	TOT	Ab	No Ab	TOT
Genotype TT	0	16	16	0	4	4	0	18	18
Genotype CT	1	12	13	0	2	2	1	18	19
Genotype CC	2	15	17	1	9	10	3	25	28
TOTAL	3	43	46	1	15	16	4	61	65

Note: Ab = patients who developed antibodies; No Ab = patients who did not develop antibodies.

**Table 4:** Contingency table: association between allelic variation in HLA-DQA1 gene and the presence of anti-drug antibodies to anti-TNFα.

ALLELE	IFX			ADA			ANTI-TNF		
	Ab	No Ab	TOT	Ab	No Ab	TOT	Ab	No Ab	TOT
T	1	44	45	0	10	10	1	54	55
C	5	48	53	2	20	22	7	68	75
TOTAL	6	92	98	2	30	32	8	122	130

Note: Ab = patients who developed antibodies; No Ab = patients who did not develop antibodies.

## Data Availability

Data will be made available on request.
